# Use of Multiple Sequencing Technologies To Produce a High-Quality Genome of the Fungus *Pseudogymnoascus destructans*, the Causative Agent of Bat White-Nose Syndrome

**DOI:** 10.1128/genomeA.00445-16

**Published:** 2016-06-30

**Authors:** Kevin P. Drees, Jonathan M. Palmer, Robert Sebra, Jeffrey M. Lorch, Cynthia Chen, Cheng-Cang Wu, Jin Woo Bok, Nancy P. Keller, David S. Blehert, Christina A. Cuomo, Daniel L. Lindner, Jeffrey T. Foster

**Affiliations:** a Department of Molecular, Cellular and Biomedical Sciences, University of New Hampshire, Durham, New Hampshire, USA; b Center for Forest Mycology Research, Northern Research Station, U.S. Forest Service, Madison, Wisconsin, USA; c Department of Genetics and Genomic Sciences and Icahn Institute for Genomics and Multiscale Biology, Icahn School of Medicine at Mount Sinai, New York, New York, USA; d U.S. Geological Survey, National Wildlife Health Center, Madison, Wisconsin, USA; e Intact Genomics, Saint Louis, Missouri, USA; f Department of Bacteriology and Department of Medical Microbiology and Immunology, University of Wisconsin, Madison, Wisconsin, USA; g Genome Sequencing and Analysis Program, Broad Institute of MIT and Harvard, Cambridge, Massachusetts, USA

## Abstract

White-nose syndrome has recently emerged as one of the most devastating wildlife diseases recorded, causing widespread mortality in numerous bat species throughout eastern North America. Here, we present an improved reference genome of the fungal pathogen *Pseudogymnoascus destructans* for use in comparative genomic studies.

## GENOME ANNOUNCEMENT

Since emerging in 2006, white-nose syndrome has rapidly spread across eastern North America from an index site in New York, causing the mortality of millions of bats of numerous species (1). The pathogen was identified as *Pseudogymnoascus destructans* (2, 3), a psychrophilic fungus that infects bats during hibernation, when bat body temperatures decrease to the ambient temperature of their hibernacula (4). The growth of this fungus on bats and subsequent invasion of epidermal tissues can cause a cascade of deleterious physiological changes resulting in high mortality rates (5, 6). The fungus is widespread in Eurasia (7–9), and initial genetic and experimental results suggest that it was recently introduced to North America (10, 11). Critically missing, however, are detailed genomic analyses of *P. destructans* to shed light on the origins, evolution, global dispersal, and pathogenicity of this fungus.

Initial genome sequencing efforts for *P. destructans* used 454 and Illumina platforms; however, unresolved repeat regions in the genome resulted in fragmented draft assemblies (12). Therefore, to generate a high-quality contiguous assembly of the *P. destructans* genome, we utilized a combination of data from PacBio SMRT reads, Illumina MiSeq 250-bp paired-end reads (SRR1952982), 8-kb 454 “jumping” libraries (SRP001346), and end sequences from an unbiased random shear bacterial artificial chromosome (BAC) library with an average insert size of 100 kb (13).

The PacBio reads were assembled with the PacBio SMRT version 2.2 analysis pipeline (14) and yielded 153 contigs. Using Illumina 250-bp paired-end MiSeq reads quality-trimmed to Q30 with ea-utils version 1.1.2 (15) and error-corrected with Hammer (16), 454 8-kb jumping reads, and >100-kb BAC library end sequences, the contigs were extended and scaffolded with SSPACE version 3.0 (17); gaps were removed with GapFiller version 1.10 (18). Twenty iterations of SSPACE/GapFiller were followed by Pilon version 1.8 (19) to correct local misalignments and indels from the assembly, resulting in 96 scaffolds. A self-query of the improved scaffolds with the NUCmer application of MUMmer version 3.23 (20) indicated that the smaller scaffolds (<42,633 bp) and a few larger scaffolds were complete or nearly complete duplications of the sequence in other scaffolds. Thirteen scaffolds, which had a >95% identity over 95% of their length with a larger scaffold in the assembly, were removed. The final assembly contains 83 scaffolds, is 35.818201 Mb in size, has an *N*_50_ value of 1.168637 Mb, and contains only 7,812 bp of gaps. This is a significant improvement compared to previous draft assemblies of *P. destructans* (AEFC01: 1,847 scaffolds, 30.6849 Mb, *N*_50_ 105.158 kb, 2,328,879 bp Ns; AYKP01: 5,304 scaffolds, 30.2827 Mb, *N*_50_ 17.914 kb, 0 Ns).

The new *P. destructans* genome contains 38.17% repetitive sequence elements (RepeatModeler version 1.08, RepeatMasker version 4.05, http://www.repeatmasker.org), which required a combination of long-read and large-insert sequencing technologies to scaffold. The nearly complete assembly presented here should serve as a valuable resource to facilitate fungal disease research.

### Nucleotide sequence accession numbers.

This whole-genome shotgun project has been deposited in DDBJ/ ENA/GenBank under the accession number LAJJ00000000. The version described in this paper is the first version, LAJJ01000000.
